# A Simple Framework for Collaborative Development of Predictive Models Trained on Proprietary Data

**DOI:** 10.1021/acs.jcim.5c02068

**Published:** 2025-11-18

**Authors:** Pablo Rodríguez-Belenguer, Alexander Amberg, Frank Bringezu, Markus Frericks, Jennifer Hemmerich, Peter Monecke, Nils Oberhauser, Panuwat Trairatphisan, Manuel Pastor

**Affiliations:** † Biomedical Imaging Research Group (GIBI230), Instituto de Investigación Sanitaria La Fe, Valencia 46026, Spain; ‡ 435921 Sanofi, Preclinical Safety, Industriepark Hoechst, Frankfurt am Main 65926, Germany; § 2792 Merck Healthcare KGaA, Chemical & Preclinical Safety, Darmstadt 64293, Germany; ∥ 5184 BASF SE, Registered Office, Ludwigshafen 67056, Germany; ⊥ 1528 Novartis Pharma, Basel CH-4056, Switzerland; # Research Programme on Biomedical Informatics (GRIB), Department of Medicine and Life Sciences, Hospital Del Mar Medical Research Institute, 16770 Universitat Pompeu Fabra, Barcelona 08003, Spain

## Abstract

We present a simple methodology that allows the building and sharing of predictive models without compromising the confidentiality of the structures of the training series. Multiple shared models can be used to obtain ensemble models, providing better coverage of the chemical space and better predictions than the original one. This approach is demonstrated in a collaborative exercise where four pharmaceutical and chemical companies developed predictive models for the AMES mutagenicity end point and shared them to build ensemble models using logical and machine learning algorithms. The results were systematically analyzed and compared, obtaining clear benefits in predictive quality. The method has the advantage of being very simple to execute, using only open-source software, and the possibility to audit the whole process and interchange files to guarantee that no confidential information is exported from the company facilities.

## Graphical abstract

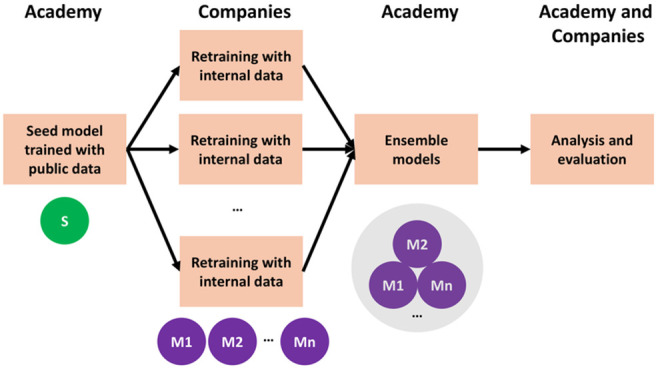

## Introduction

Predictive models have been demonstrated to be a valuable tool for exploiting the growing volume of experimental data generated in pharmaceutical research.^1,2^ Models are being regarded as valuable digital assets, able to generate useful information that can be incorporated into decision-making workflows at candidate design and development. However, their value is tightly associated with the quality of the data used to train these models. In particular, predictions can only be considered reliable when the models are applied to substances within the model applicability domain.^3,4^ For this reason, using large training data sets covering wide areas of the druggable chemical space is one of the most critical factors determining the model’s usefulness.

The IMI eTRANSAFE project recently demonstrated the possibility of establishing effective precompetitive research collaboration, sharing large volumes of drug safety information (over 10,000 studies).^5^ In the context of this collaborative effort, it was also decided to explore the possibility of sharing raw toxicity data from collections of compounds annotated with experimental information, typically used to build in-house predictive models, to generate many large data sets. The potential benefit of this initiative is clear: models trained on series donated by multiple companies will provide better coverage of the chemical space and generate more reliable predictions. From the companies’ standpoint, this exercise would allow them to enrich the information included in their individual models at no additional cost, covering unexplored regions of the chemical space that could be of particular interest for predicting the properties of novel candidates. As the biological annotations of this initiative describe drug safety information, this exercise fits perfectly into the precompetitive modeling environment since such a collaboration would not compromise the innovation generated by any single company but would rather enhance the collective safety of their drugs.

In its simplest form, the practical implementation of this collaboration would require a compiling of all the chemical structures and their annotations in a single place for training the joint model. This approach is unfortunately unfeasible since many of the structures are confidential. Sharing them requires a complex verification and approval procedure and usually occurs only between noncompetitors in very limited substance exchanges. With this aim, we describe here innovative methods that allow sharing predictive models without exposing chemical structures.

To build models for collaboration in computational toxicology without compromising data confidentiality, innovative federated learning methods have been developed to share predictive models without disclosing chemical structures. The MELLODDY project stands out as a milestone in this field, involving 10 pharmaceutical companies in an unprecedented effort. Using a data set of over 2.6 billion confidential experimental activity data points, covering 21+ million small molecules and 40,000 assays, MELLODDY implemented a federated multitask learning approach. This method allowed each company to improve its own classification and regression models without compromising proprietary information.^6^ Similarly, the Effiris platform, developed by Lhasa Limited, employs a different federated learning scheme where an initial model is trained on public data and then refined with internal data from multiple partners.^7^ The FL-QSAR project demonstrated the feasibility of horizontal federated learning for Quantitative Structure–Activity Relationship (QSAR) modeling, enabling pharmaceutical institutions to collaborate on QSAR analysis without sharing sensitive compound data. This approach not only outperformed single-client models but also achieved performance comparable to cleartext learning algorithms using all shared information.^8^ These projects collectively showcase the potential of federated learning strategies in drug discovery and toxicity prediction, demonstrating improved predictive performance, extended applicability domains, and the ability to leverage collective training data volumes, including auxiliary data from high-throughput assays, while strictly maintaining data privacy and security. Nevertheless, these approaches also require complex computational infrastructure, sophisticated software, as well as rigorous data governance policy which might hinder smaller fit-for-purpose collaboration efforts with a similar goal

In this work, we describe a new strategy for the collaborative development of predictive models without sharing training series based on simple machine learning (ML) methods. The whole procedure is extremely simple and fully auditable, and the final models can be used locally with a simple open-source tool. The application of the method is illustrated in a sharing exercise involving four companies (BASF, Merck Healthcare KGaA, Novartis, and Sanofi) where it was used to develop a model for AMES test, an *in vitro* bacterial bioassay to evaluate the mutagenicity of pharmaceutical and chemical substances.^9^ The quality of the resulting ensemble models is illustrated and compared to the individual models, using a systematic validation exercise that was designed to produce realistic results. The results confirm that the ensemble models capture valuable information from all the models and can provide slightly better predictions than the best individual model and much better predictions than the worst ones. Furthermore, the ensemble algorithms can be easily tuned to optimize the sensitivity or specificity of the results according to the intended model usage, e.g., for screening at an early stage versus as a confirmatory testing at a later stage of drug development.

## Methods

### Data Sets

For the development of confidential models, the four companies extracted a series of compounds annotated with their AMES test results performed as GLP or GLP-like studies from their internal data repositories. No common criteria were imposed for curating the structures of this internal data. The results of the AMES test were annotated as positive or negative for each compound. The training series used for this modeling exercise consisted of a single SDFile per company, with the AMES annotation included as a field of the file. These files were used internally for training the models and were never shared with any other partner nor sent outside of the company’s facilities.

For the validation of all the ensemble models and the training of ensemble models built using ML, we need an additional data set, the so-called common validation data set (CVD). The first version of this series was obtained from a literature search. The annotated chemical structures were curated in a workflow that included removal of incomplete or confusing records, such as inorganics, organometallics, counterions, biologics, and mixtures.^10^ Further steps involved structural cleaning (e.g., detection of valence violations), ring aromatization, the normalization of specific chemotypes, and the standardization of tautomeric forms. These tasks were automated using Chemaxon InstantJchem.^11^ After data curation we obtained and comprised 10,264 compounds, 5,503 positives (mutagenic compounds), and 4,761 negatives (nonmutagenic compounds). However, to validate the models fairly, we need to ensure that no compound in this series is also present in the internal training series used by the companies. This task was carried out by individual companies due to data confidentiality by locally running a script on the CVD to remove any compound present in their internal training series. To guarantee that these compounds were identified and removed, the script used at least one of the two following criteria:

1. InChIKey Matching: The function first checks for matches between the InChIKey^12^ of compounds from the CVD and each company’s training series. In the InChIKey matching, we intentionally did not consider the last two characters to disregard the structure’s ionization status.
2. Tanimoto Similarity Threshold: If there is no exact match, the function computes the Morgan fingerprints^13^ (nbits = 2048, radius = 2, features = enabled) and calculates the Tanimoto similarity coefficient between the compounds. A similarity threshold of 0.95 was applied to identify compounds in the CVD which were considered equivalent for the purposes of this analysis.

If either of these tests detects that the compound is present in the company training series, a found flag is assigned to that compound, indicating it is present in both data sets. The Universitat Pompeu Fabra (UPF), as the honest broker, collected and consolidated the results of running this script by the four companies, obtaining a final version of the CVD with 2,442 compounds with 1,048 positives, and 1,394 negatives. One could argue that this procedure may lead to the identification of internal training compounds by comparing the CVD before and after the cleanup. However, it should be noted that this procedure removes only compounds already present in the original CVD version, which was created from bibliographic sources, and containing no proprietary structure. So, in the worst-case scenario, it can be identified that some companies included compounds found in the bibliography in their training series. However, this is not a threat for the confidentiality of their proprietary substances.

### Model Building

QSAR models were built with the Flame 1.2.2 ^14^ modeling framework and trained on series of compounds with a binary mutagenicity annotation derived experimentally from the AMES test. Data were derived from AMES test on the following microbial strains: Salmonella typhimurium TA97a, TA98, TA100, TA102, TA1535, TA1537, TA1538 and/or *E. coli* WP2, WP2uvrA. Random undersampling was applied to the majority class to address potential class imbalance, ensuring balanced proportions for both classes. The training series structures were preprocessed using a standardizing script [https://github.com/flatkinson/standardiser] for normalization. Models used Morgan fingerprints (FPs) as molecular descriptors, calculated using the RDKit library (2020.09.1.0), as implemented in Flame, with default parameters (nbits = 2048, radius = 2, features = enabled). These descriptors were scaled using the StandardScaler algorithm from scikit-learn version 0.24.1,^15^ which transforms the features to have a mean of zero and a standard deviation of one. The SelectKBest algorithm from scikit-learn was used to select the best features, with the number of top features (*K*) set to 500. The models were built using Partial Least Squares Discriminant Analysis (PLS-DA).^16^ All models were trained using a grid search with 5-fold cross-validation (CV) to find the best number of latent variables and threshold discriminating positive from negative class for the final version of the models. The models being trained by the individual companies are termed “local models”. All these models have been uploaded to a public repository (see Data Availability section) from where they can be downloaded and imported into open-source Flame software. When used for building ensemble models each local model was named “low-level models” while the combined models generated from models from multiple companies are termed “high-level models” or “metamodels”. The ensemble models can be imported into Flame and used to predict the mutagenic properties of new compounds.

The predictive performance of all models was assessed by computing sensitivity, specificity, and the Matthews correlation coefficient (MCC) (eqs 1, 2, and 3, respectively) in a 5-fold CV exercise.

1 $$\mathrm{Sensitivity}=\frac{\mathrm{TP}}{\mathrm{TP}+\mathrm{FN}}$$

2 $$\mathrm{Specificity}=\frac{\mathrm{TN}N}{\mathrm{TN}+\mathrm{FP}}$$

3 $$\mathrm{MCC}=\frac{\mathrm{TP}\times \mathrm{TN}-\mathrm{FP}\times \mathrm{FN}}{\sqrt{\left(\mathrm{TP}+\mathrm{FP}\right)\left(\mathrm{TP}+\mathrm{FN}\right)\left(\mathrm{TN}+\mathrm{FP}\right)\left(\mathrm{TN}+\mathrm{FN}\right)}}$$

In the three equations above, TP, TN, FP, and FN mean the number of true positives, true negatives, false positives, and false negatives, respectively.

### Confidential Model Building

Flame software implements an innovative feature that guarantees that the model can be shared without disclosing the structures of the training series. If this option is activated during the model-building stage, the features’ scaling information (standard deviation and mean) and the model coefficients for each of the model features are saved as human-readable text files. These two text files and a modeling parameter file can be compressed into a single export file, which contains the bare minimum information to generate predictions for new compounds. This file can be shared with confidence since it contains no trace of the training series and can be easily audited. The Flame software can import confidential models shared by exchanging the export file by e-mail or other digital interchange methods, generating a confidential model that is capable of producing predictions for new compounds.

The confidential status of the model is shown in the Flame interface. Models built in confidential mode, and thus able to generate confidential export files, are shown with an open lock, while confidential models, obtained after importing a confidential export file, are shown with a closed lock (see Figure 1).

**Figure 1:**
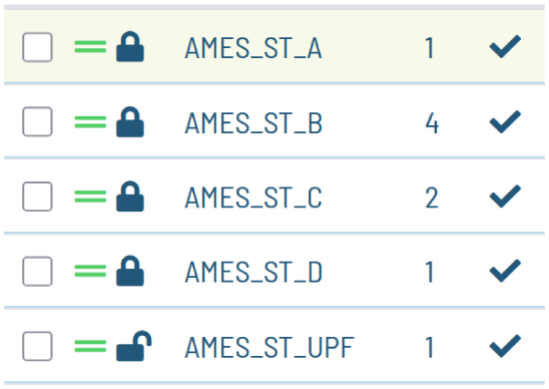
Detail of the Flame GUI interface, showing the list of models and an icon which indicates if the models have been built in confidential mode (open lock) or are imported as secret models (closed lock).

In contrast to regular/nonconfidential models, confidential models cannot show any depiction of the chemical space, and they lack other features that would require access to the training series. It must be noted that this information from the training series is not masked or encrypted and is not accessible either because it is not included in the export file and never leaves the computer where the model was originally built. This confidential model building feature is only available for models built using Partial Least Squares, since the model coefficients were simpler to extract for these methods.

Although no information about the training series structures is shared, confidential models have access to the mean and standard deviation of each variable used by the model. This aggregate information can be used to generate approximate visualizations comparing the regions of the chemical space covered by a predicted series and each of the training series used by a metamodel. The method consists of generating several synthetic objects equal to the size of the original training series, represented by variable values drawn from a normal distribution generated using the stored means and standard deviations. It must be stressed that these synthetic objects consist only of arrays of molecular descriptors, with no associated molecular structures. Then, when using the ensemble model for predicting a series, the descriptors generated for each of the individual series are used to build a Principal Component Analysis (PCA) model, on top of which we can project the synthetic objects representing the region covered by the training series. Figure 2 depicts an example of the graphic generated in Flame, where the compounds in the predicted series are shown as red (positive) and blue (negative) dots, while the synthetic objects representing the training series are shown as transparent green circles. It shall be emphasized that this representation is only approximated and cannot be expected to capture the fine details of the original training series distribution. In particular, internal testing showed that the spanning of the synthetic objects tends to be slightly narrower than the original compounds. The graphic in Figure 2 shows how the training series provides good coverage of the center of the series, while the dots scattered on the left-hand and right-hand regions are not so well represented. Since the graphic is interactive, it is possible to show the structures of single compounds or clusters of compounds by selecting them with the mouse on the screen. This feature facilitates identifying the structure of compounds that are likely to be out of the model applicability domain because they are far from the region covered by the training series.

**Figure 2:**
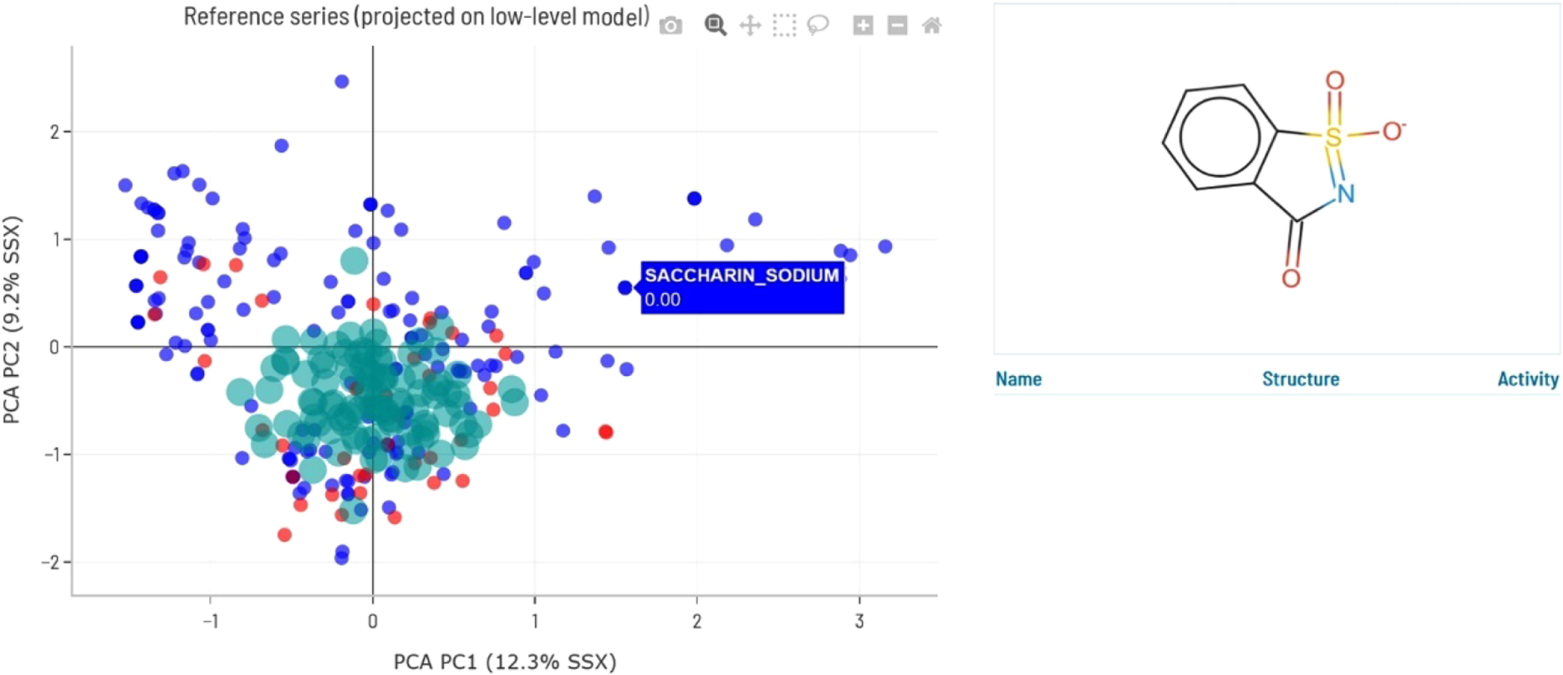
PCA scores plot representing a chemical series predicted by a confidential ensemble model. The PCA was computed using the molecular descriptors of the predicted series for each one of the low-level models. Red points correspond to AMES-test positive compounds and blue points to AMES-test negative compounds. Green dots are synthetic objects generated to highlight the region of the chemical space covered by the training series of confidential low-level models. In Flame, this graphic is interactive and allows the structure of compounds selected with the mouse to be shown.

### Model Combination

The local models developed by the companies were trained using binary annotations and produced binary predictions. The first approach to obtain integrated predictions is to combine the results of all the models using logical rules, such as the Logical OR: Positive when any of the four models produces a positive prediction and negative otherwise, or the Majority voting: Positive when the number of models producing a positive prediction is higher than the number of models producing a negative result and negative otherwise. To further clarify the application of these rules in this exercise, we have shown in Table 1 the result obtained depending on the number of positive predictions obtained.

**Table 1:** Results Obtained by the Ensemble Models Using Logical OR and Majority Voting, Depending on the Number of Positive Predictions Obtained by the Low-Level Models

#Positive predictions	Logical OR	Majority voting
0	Negative	Negative
1	Positive	Negative
2	Positive	Negative
3	Positive	Positive
4	Positive	Positive

It should be noted that ensemble models built using logical rules do not require any training, and their results are obtained directly from the predictions of the low-level models.

In the second stage of the study, the individual predictions obtained from the low-level models were used as input variables to train a new high-level model. In this study, we used the following machine learning methods: Random Forest (RF), Support Vector Machines (SVM), and XGBoost. The training series used was the CVD described above, containing 2442 annotated compounds.

### Model Validation

As an integral part of the model development, the predictive quality of individual models was initially validated using 5-fold cross-validation. To report model quality or other company-specific details, company names were randomly anonymized as A, B, C, and D.

Additionally, the predictive quality of most models developed in this exercise was assessed using an external data set. The CVD was used for this purpose whenever possible since it contains a good collection of annotated compounds not used in any of the individual confidential models. This data set also facilitates the comparison of the results approaches using different methodologies.

The industrial partners further evaluated the predictive quality and usefulness of the best metamodels, applying metamodels developed ad hoc for this analysis by removing their own training series. This means that, for this test only, we used four different types of ensemble models, each built with data from other companies but without the data of the company performing the test. In each instance, we developed two models: logical OR and majority voting. These models were used to predict internal series of compounds, but they were not used in the training of their own models. The size of the test series was 1.087 compounds for A, 2.265 for B, 59 for C, and 3.112 for D.

## Results and Discussion

### Overview

The present study is a collaborative modeling exercise where four companies developed QSAR models using series of internal compounds. These models were built and shared as confidential models without disclosing any training series information, and they were used for building a set of metamodels. The hypothesis is that these metamodels have better predictive quality and can be more useful than any of the individual models. This was tested by using the metamodels in two different validation exercises: one predicting test series extracted from the literature (CVD), and another predicting test series of internal compounds from the companies. Figure 3 illustrates the different steps of the exercise.

**Figure 3:**
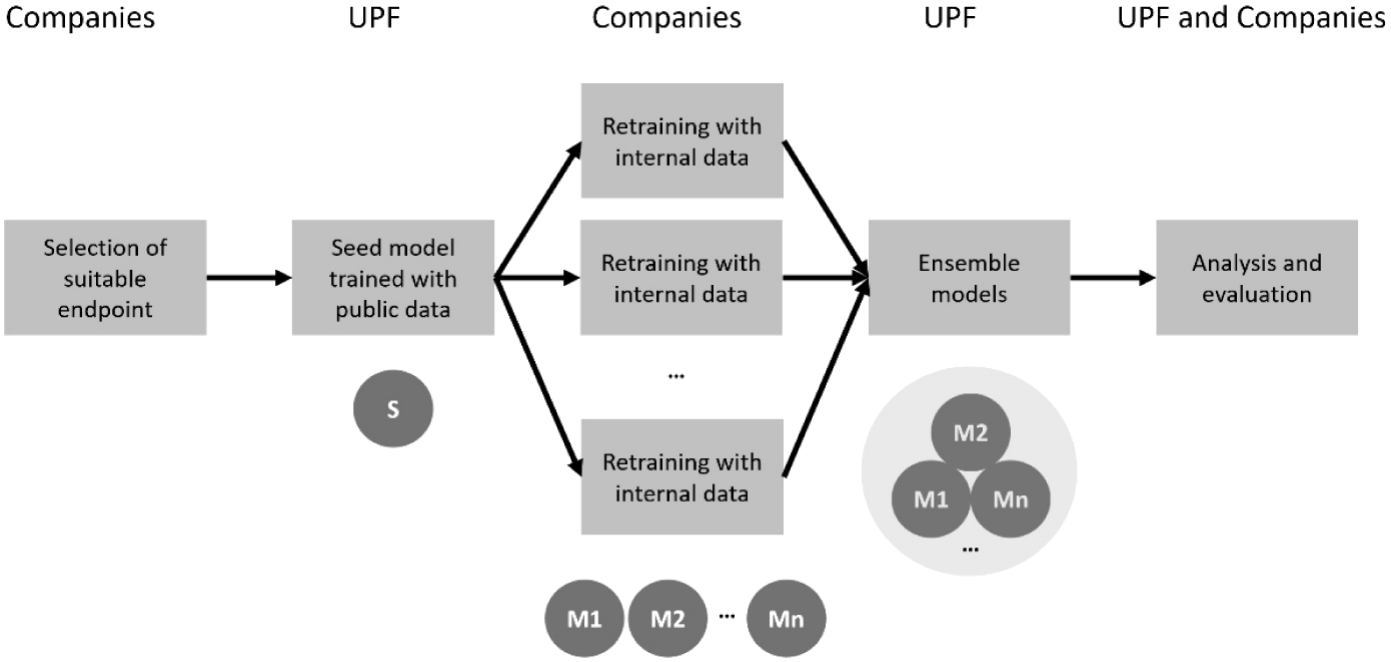
Overview of the different steps of the study. After selecting a suitable end point, a seed model S was sent to the companies, who retrained it with a confidential series to generate local models M1 to Mn. These were collected and used to build different ensemble models that were analyzed and evaluated systematically.

### Endpoint Selection

The four companies participating in this exercise (BASF, Merck Healthcare KGaA, Novartis and Sanofi), all partners of the eTRANSAFE project, were consulted about suitable toxicological end points for collaborative modeling exercise. The criteria were that the end point should be of interest for all participants, the company should have sufficient data (obtained by themselves or from contract research organizations) which are accessible and generated for internal compounds, and the experimental methods used for generating this data should be comparable across participating companies. A list of candidate end points is listed in Table 2

**Table 2:** End Points of Interest

AMES mutagenicity test
Phospholipidosis
Micronucleus test
Mitochondrial toxicity (Glu/Gal)
Cytotoxicity (HepG2)
Cytotoxicity (PBMC)
Kinase inhibitor models
Cytotoxicity primary hepatocytes
Glutathione (GSH) trapping
Mechanism based inhibition (MBI)

After discussion, the AMES mutagenicity end point was selected as the best compromise solution for all the aforementioned criteria. Furthermore, AMES mutagenicity test is required by regulatory submission, it has a well-established experimental protocol, and the interpretation is simple and straightforward i.e., positive vs negative results. It is worth commenting that to apply this methodology all companies must agree on the end point to be predicted and therefore, the target involved, and the details of the test must be disclosed. This may limit the application of the procedure for proprietary projects where the targets themselves could be confidential.

### Development of Individual Models

The companies generated the training series using their internal data (see the Methods section for details) and built their confidential models. To ensure that the models are trained in comparable conditions, an initial (so-called “seed model”) model was developed by the leading academic partner (UPF) using a series of 206 compounds extracted from ChEMBL, 80% negative (165) and 20% positive (41). The model was built in Flame using the general procedure described in the Methods section: Morgan Fingerprints and Partial Least Squares-Discriminant Analysis (PLS-DA) with simple subsampling, producing a final training series of 41 positive and 41 negative compounds. It should be emphasized that this choice of descriptors and machine learning algorithm was oriented to obtain comparable models by all the participating companies with their own data and not optimized for obtaining the best results. In real-world applications, no comparison of the initial model quality would be intended. Hence, the use of a seed model is not required for such cases.

This model was imported by the four companies and retrained with their own training series using the same modeling settings, obtaining four different individual models. After resampling, the sizes of the training series were 6894 for A, 1098 for B, 1010 for C, and 1232 for D, (see Table 3).

**Table 3:** Sizes of the Training Series and Predictive Quality of Individual Models, Obtained by 5-Fold CV

Company	#Compounds	Sensitivity	Specificity	MCC
**A**	6894	0.79	0.82	0.60
**B**	1098	0.88	0.60	0.50
**C**	1010	0.76	0.67	0.43
**D**	1232	0.80	0.75	0.55

It must be noted that applying this procedure (retraining a common model with internal data) guarantees that all models were built using the same descriptors, machine learning algorithm, and resampling strategy. This method also builds the models in confidential mode (see Methods section), allowing to export them as “secret models”: only as PLS coefficients plus the statistical information (mean and standard deviation) of the fingerprint’s matrices.

The size of the training series after the undersampling and predictive quality of the models (obtained using a 5-fold CV) are shown in Table 3. In terms of sensitivity, the ability to correctly identify mutagenic compounds ranged from 0.76 (Company C) to 0.88 (Company B), indicating that all models effectively detected mutagenic compounds. However, specificity—the ability to correctly classify nonmutagenic compounds—varied more significantly, with Company B achieving a specificity of 0.60 while Company A performing notably better with a specificity of 0.82. This variation in specificity suggests that some models, particularly the one from Company B, tended to misclassify nonmutagenic compounds as mutagenic more frequently.

Company A’s model obtained the highest MCC at 0.60, indicating a strong overall performance, while Company C’s and Company B’s models had lower MCCs of 0.43 and 0.50, respectively. This suggests that Company A’s model has a better trade-off between identifying true positives (mutagenic compounds) and avoiding false positives (nonmutagenic compounds), likely benefiting from the larger training set.

The predictive quality of these individual models was further assessed by using them to predict the compounds in the CVD as an external validation data set. The results are shown in Table 4.

**Table 4:** Predictive Quality of Individual Models Obtained Using External Data Set

Company	Sensitivity	Specificity	MCC
**A**	0.73	0.74	0.47
**B**	0.79	0.47	0.27
**C**	0.36	0.87	0.27
**D**	0.64	0.72	0.36

As shown in Table 4, the individual models performed poorer on the external validation set with sensitivity values ranging from 0.36 to 0.79 and specificity values ranging from 0.47 to 0.87. Remarkably, the results obtained with this external data set do not reproduce the CV results, which could be justified by the different levels of overlap between the regions of chemical space covered by the validation series and the models’ training series. Company A’s model obtained the best overall score, with rather balanced sensitivity and specificity, obtaining an MCC of 0.47. Nevertheless, even if MCCs from Company B’s and Company C’s models are the lowest, they have the highest sensitivity and specificity values within the group, respectively. These results highlight the potential advantages of complementing the models generated by one company with additional information provided by external companies.

### Development of Ensemble Models

The models obtained by the companies were exported as confidential models as explained in the Methods section, and they were sent to the academic partner (UPF) for building ensemble models, as shown in Figure 3.

We would like to emphasize that each model contains knowledge about the particular region of the chemical space from each company, which corresponds to the training series being extracted. The main value of this exercise is to combine this knowledge into ensemble models. According to our initial hypothesis, these ensemble models would have better predictive performance than the original individual models. Therefore, the next step is to investigate the best approaches for obtaining these ensemble models so that we can later analyze their predictive quality and verify our hypothesis.

Individual QSAR models can be combined using different methods, as presented in the Methods section. We started by building ensemble models using logical rules (logical OR and majority voting). The predictive quality of the models was assessed by predicting the CVD, obtaining the results shown in Table 5.

**Table 5:** Predictive Quality of Ensemble Models Obtained Using Logical Operators

Method	Sensitivity	Specificity	MCC
logical OR	0.94	0.36	0.36
majority voting	0.70	0.81	0.51

In terms of overall quality, the majority voting model exhibits good predictive quality, with an MCC higher than any of the individual models (0.51 versus 0.47, see Tables 4 and 5). In a practical model-sharing exercise, all companies would see a large improvement in the predictive performance, with the only exception of Company A, for which the improvement is only modest due to better specificity (from 0.74 to 0.81), while the sensitivity of the ensemble model is slightly lower (from 0.73 to 0.70). On the other hand, if the goal is to develop an early in silico alerting system, the logical OR model shows an impressive sensitivity of 0.94, even if the specificity is so low that an experimental assay should confirm the positives. All in all, these two examples show how the individual models can be combined to obtain better predictions, emphasizing either the sensitivity or the specificity but also improving the overall predictive quality with respect to the individual models.

Another approach for integrating the individual models uses high-level machine learning methods to combine the prediction results produced by the individual models. Unlike the previous example, this approach required training a high-level model, using as predictors the results produced by the individual models. Further details about how these models were built are provided in the Methods section.

For model training, we used the CVD as input data since this series does not contain any of the compounds represented in the low-level models. However, it should be noted that the simplicity of the input data (only four variables with binary input) makes the use of the same compounds for training and prediction irrelevant since there are only 2^4^ (16) input values, and the training process only identifies the optimal way to combine them to fit the observed data. The results obtained using three different ML algorithms are shown in Table 6.

**Table 6:** Quality of Ensemble Models Obtained Using ML Algorithms

Method	Sensitivity	Specificity	MCC
**RF**	0.73	0.74	0.47
**SVM**	0.73	0.74	0.47
**XGBoost**	0.73	0.74	0.47

As we can see, all the algorithms yield exactly the same results, showing that the problem of combining this limited variability of inputs is not challenging, and all of them converge on the same profile. In all cases, we obtained high-level models with rather balanced sensitivity and specificity, which can be considered an intermediate between the logical OR and the majority voting, with slightly better sensitivity but slightly worse specificity than the majority voting model. Even if the MCC is lower than the one obtained for the majority voting model, these models provide a compromise between sensitivity and specificity, which can be the most useful option in the absence of strong requirements in one sense or the other.

At this moment of the study, we can start discussing the advantages of the ensemble models. The results in Tables 5 and 6 indicate that ensemble models can produce better predictions, according to generic parameters like MCC, and there is a possibility to tune the models to obtain either more sensible or more specific results. Also, a closer inspection reveals that not all companies would benefit from the same level of improvement. For example, Company A, which contributed the largest data set, would benefit from the ensemble model only modestly, while other companies would obtain a much larger quality boost from model sharing. These differences can be better appreciated in Figure 4, where we represent the changes in sensitivity and specificity obtained when moving from the local model to the majority voting or ML (RF) ensemble models.

**Figure 4:**
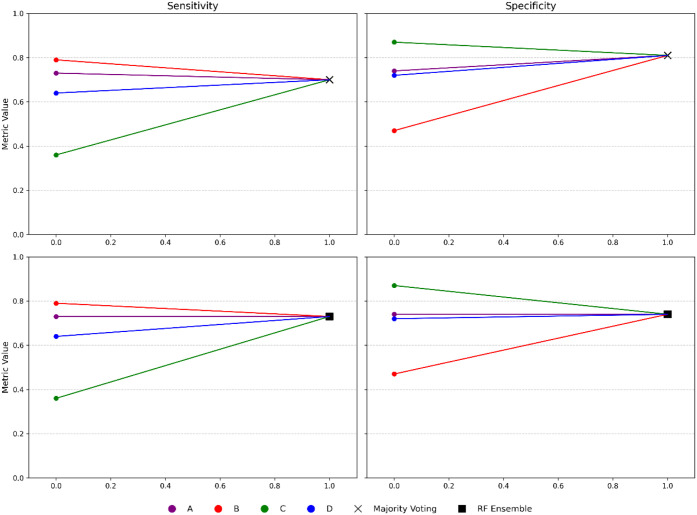
Change in sensitivity (left panel) and specificity (right panel) were obtained when using a majority voting (top) or RF (bottom) ensemble models, compared with the individual company models.

The top graphics of Figure 4 clearly showed how the Majority voting ensemble model has better sensitivity than some individual models (C and D) and better specificity than the individual models (A, B, and D). A similar effect can be observed for the RF ensemble model, even if, in this case, the sensitivity and specificity of the ensemble are more balanced.

It should also be noted that the prediction quality indexes used in this analysis probably fall short of characterizing the benefits obtained for the prediction of original structures, which are poorly represented in the internal data sets and represent precisely innovative chemicals of high potential value for the companies. Unfortunately, due to the characteristics of this exercise, we have been unable to quantify this potential benefit, which is mentioned here as a plausible hypothesis.

Another aspect in which this collaboration exercise can contribute to an improvement is the chemical coverage of the models. A direct representation using the compounds used in the training series is not feasible because this information was omitted in the confidential models. However, the visualization tool implemented in Flame, described in the Methods section, offers an interesting alternative for visualizing the chemical space covered by the training series of individual companies and comparing it with a predicted series. In Figure 5, we represented the chemical space overlapping of the CVD with synthetic objects, estimating the chemical space covered by the low-level models.

**Figure 5:**
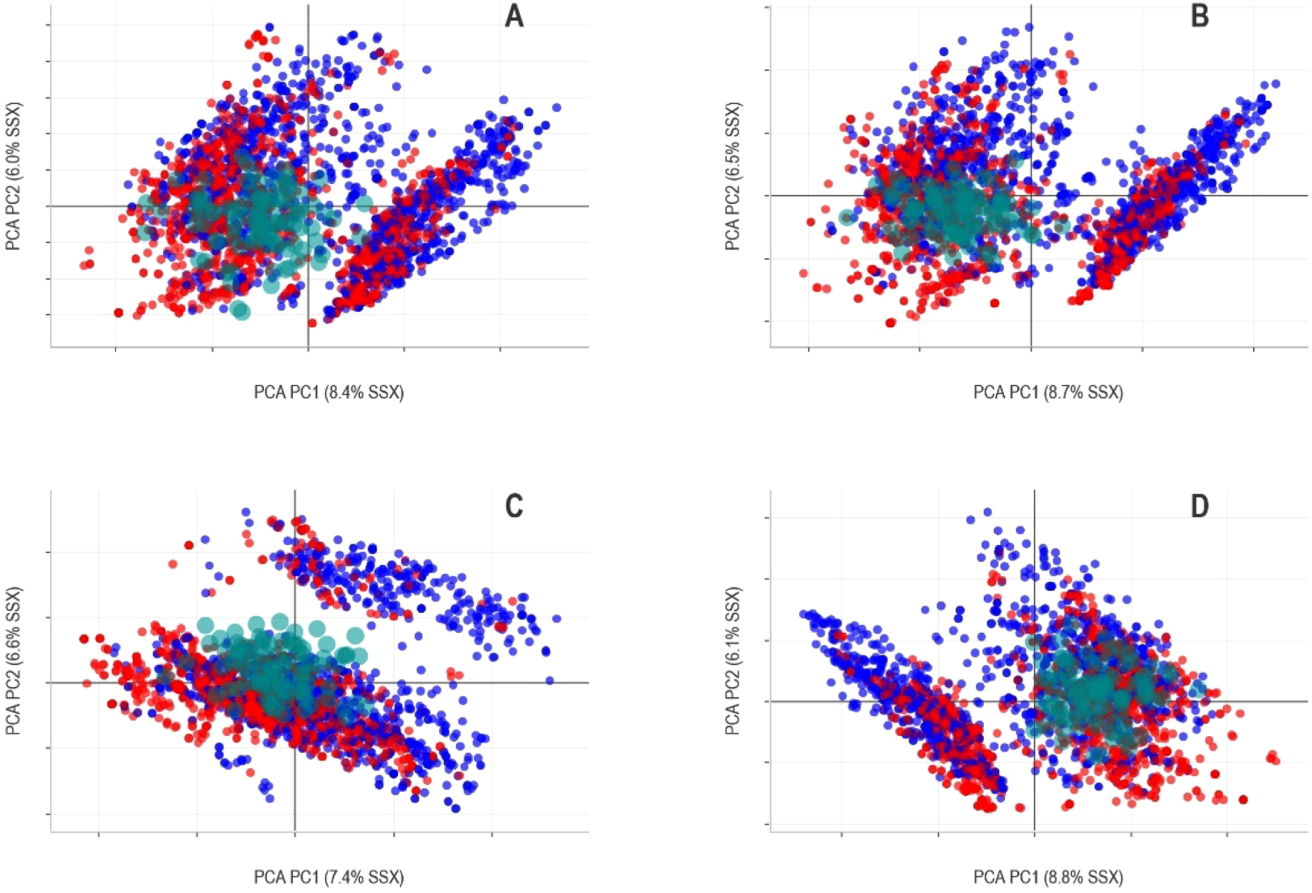
PCA scores plot representing series CVD and synthetic objects. Red points correspond to positive compounds and blue points to negative compounds in CVD. Green dots were synthetic objects generated to highlight the region of the chemical space covered by the training series of low-level models produced by the different companies (A, B, C, D).

Interestingly, we can see how the low-level models with higher overall predictive quality based on MCC (A, B, and D) provide a higher chemical overlapping. In contrast, the training series of the C model covers a narrower region, with some clusters rather separated from the training series. Still, this graphic should be interpreted with caution because the two first principal components (PC) represent only a small part of the information (approximately 15% of the sum of squares) present in the descriptor matrix of the predicted series.

### Validation with Internal Series

As a last step in systematically validating the proposed methodology, we tried to simulate the application in practice of the best ensemble models. With this aim, we developed separate versions of the ensemble models for each company by removing their own individual models (see Methods section), called cutout models. This guarantees that the predictions obtained with a company ensemble will not produce overoptimistic results due to the presence of a model trained with internal compounds. Besides, these cutout models shall represent the added knowledge contributed by other companies, which were not masked by the value of the already available information. In a real exercise, internal compounds would be added to the ensemble models, thus producing results closer to those in Tables 5 and 6.

The cutout models were returned to the companies and used to predict a new series of annotated compounds. Unfortunately, at this late stage of the project, some companies had difficulties obtaining additional compounds, and the validation series were generally smaller than the original training series. The results obtained are shown in Table 7.

**Table 7:** Validation of Ensemble Models Using Internal Validations Series

Company	#Compounds	Method	Sensitivity	Specificity	MCC
A	1087	Logical OR	0.91	0.32	0.11
B	2265	Logical OR	0.89	0.39	0.25
C	59	Logical OR	1.00	0.28	0.10
D	3112	Logical OR	0.93	0.29	0.20
A	1087	Majority voting	0.49	0.79	0.15
B	2265	Majority voting	0.67	0.71	0.33
C	59	Majority voting	1.00	0.60	0.16
D	3112	Majority voting	0.62	0.74	0.30

In general, these results resemble the ones obtained from the complete ensembles and the CVD data set. Predictions obtained using the logical OR ensembles in this validation show high sensitivity and low specificity, with values similar to those shown in Table 5 with slightly lower sensitivity and specificity values in general but with one exception of a slightly higher specificity for Model B (0.39 vs 0.36). The results of the Majority voting ensemble for this validation exercise were only slightly worse for Company B’s and Company D’s data sets: sensitivities of 0.67 and 0.62, versus 0.70 of the complete ensemble/CVD and specificities of 0.71 and 0.74 versus 0.81 of the complete ensemble/CVD. However, the model prediction quality was much poorer in the Company A’s case. This can be explained by the removal of the large amount of Company A’s data present in the complete ensemble which affects largely the sensitivity, probably by the presence of original compounds. Even so, the effect seems to affect only slightly the specificity (0.79), which is very close to the values obtained with the full ensemble/CVD (0.81). Note that the results from Company Cs are better in terms of sensitivity but worse for specificity and MCC which could be largely influenced by the much lower number of compounds in the validation set.

### Comparison of Our Current Collaborative Modeling Approach to Federated Learning

Federated learning has become a cornerstone for enabling collaboration in predictive modeling while preserving data confidentiality, particularly within the pharmaceutical sector.^8^ Our approach provides a simpler yet effective alternative to the more complex federated learning initiatives, such as MELLODDY and similar projects, which aim to leverage confidential data sets from multiple companies without sharing raw data, thus expanding models’ predictive power and applicability. Unlike MELLODDY, which employs sophisticated deep learning models that require significant computational resources, secure data encryption, and iterative synchronization across partners, our methodology is designed to be more accessible, requiring less computational overhead. We utilize simpler model building techniques, such as PLS-DA, making our approach suitable for smaller collaborations or those with limited infrastructure.

While MELLODDY maintains data decentralization and relies on real-time federated training, our approach allows each partner to independently build models that are subsequently combined using a posthoc ensemble framework. This sequential integration avoids the complexities of real-time synchronization and reduces dependency on secured communication channels.

From an auditability perspective, federated learning initiatives often face challenges in transparency due to the inherent complexity of deep learning and distributed frameworks. In contrast, using simpler models like PLS-DA ensures full auditability, enabling participants to understand feature contributions and trace decision-making processes, which is particularly beneficial for regulatory compliance. Our methodology is also highly adaptable; although we demonstrate its feasibility using PLS-DA, it can easily accommodate more complex models like Random Forests or Support Vector Machines, depending on the collaborative needs and resources available. This adaptability allows our framework to evolve incrementally, starting with interpretable models and expanding into more sophisticated approaches as capacity grows.

In summary, our methodology offers a practical alternative to federated learning for scenarios with limited infrastructure, emphasizing simplicity, transparency, and adaptability. In contrast, MELLODDY and similar federated learning projects excel when large-scale, resource-intensive collaborations are needed, where dynamic, high-performance learning and comprehensive integration of diverse data sets are critical.

### Comparison with Profile-QSAR

Another relevant initiative is Collaborative Profile-QSAR (pQSAR),^17^ which has been proposed as a natural platform for building collaborative models among competing organizations. From the perspective of confidentiality guarantees, pQSAR relies on the sharing of black-box single-assay models: each partner trains models on internal assays and distributes them in executable form, so that other participants can obtain predictions but cannot access the underlying training data or model coefficients. This provides strong protection against disclosure of raw structures or activities, but it also reduces auditability, since partners cannot examine how predictions are generated or assess feature contributions. In contrast, our framework exports only model coefficients and simple statistical summaries of descriptors (means and standard deviations). These files contain no structural information but remain fully auditable, allowing participants and regulators to understand exactly how predictions are derived. Thus, while both approaches ensure data confidentiality, the balance between opacity and interpretability differs significantly.

With respect to the role of overlap in predictive gain, both pQSAR and our framework highlight the importance of complementarity between partners’ chemical series. In pQSAR, the benefits were distributed asymmetrically, with smaller contributors gaining more than larger ones. In our collaborative exercise, however, the results were more balanced across partners: the ensemble models provided improvements of similar magnitude for all companies, without the strong dependence on data set size reported in pQSAR.

Finally, regarding scalability across end points and tasks, pQSAR has demonstrated broad applicability to thousands of assays, leveraging its flexible profile-based representation to support diverse pharmacological and toxicological end points. However, this scalability comes at the cost of maintaining and managing a large number of black-box models for each assay, as well as implementing profile filtering strategies (e.g., Max2) to reduce dimensionality. In contrast, our methodology is intentionally lightweight and end point-specific: it requires only the export of coefficients from relatively simple models, which can then be combined into ensembles without the need for large-scale infrastructure. This makes it particularly suitable for smaller collaborations or settings where rapid deployment and auditability are prioritized, while still offering a path to extension across multiple end points.

## Conclusions

In this article, we presented a methodology that can generate even more valuable models, covering wider regions of the chemical space, by sharing the learning from resources across companies. Our approach is based on a simple-to-implement, yet understandable, and auditable procedure. Our work guarantees beyond any doubt the confidentiality of any sensitive information involved because this information on chemical structures was not shared at all.

The usefulness of this approach was illustrated by applying the method in a real collaborative modeling exercise between four pharmaceutical and chemical companies, comparing the predictive quality of the individual models with ensemble models obtained by combining all the models in different ways. In general, ensemble models produce slight overall improvements with respect to the best individual models, and also very significant improvements in comparison with the worst individual models. The improvement obtained shows some inverse correlation with the size of the training series (the larger the size, the less improvement obtained). Besides, it can also be anticipated by observing the overlapping of the chemical spaces, which can be visualized using the specialized tools described here.

All in all, the results show that the ensemble models from our collaboration can produce significant predictive quality improvements for some companies. Furthermore, different ensemble modeling approaches allow us to tune the sensibility and specificity of the prediction and apply the models for different purposes. The individual models described in this article have been uploaded to a public repository (see Data Availability section) and can be downloaded, imported into Flame and used for the prediction of new compounds. All the software required for the application of this procedure on other projects is distributed free of charge and is accessible via the following address: https://github.com/phi-grib/flame.

## Data Availability

The individual models mentioned in this article were uploaded to Zenodo public repository (10.5281/zenodo.16963345). The Flame software is open source distributed under GNU GPL v3 license, accessible here: https://github.com/phi-grib/flame. Any data and associated files used by individual companies for training their respective local confidential models will remain strictly confidential and are not available for any third-party access.

## Notes

Ethical Standards: The manuscript does not contain clinical studies or patient data.
